# Comparative effectiveness of electro-acupuncture plus lifestyle modification treatment for patients with simple obesity and overweight: study protocol for a randomized controlled trial

**DOI:** 10.1186/s13063-015-1046-x

**Published:** 2015-11-17

**Authors:** Zishan Gao, Zhi Yu, Zhi-Xiu Song, Cai-Rong Zhang, Yao-Shuai Wang, Yun-Feng Wu, Bei Zhou, Shu-Ping Fu, Hao Chen, Ying Xiong, Yi Yang, Bing-Mei Zhu, Bin Xu

**Affiliations:** Second School of Clinical Medicine, Nanjing University of Chinese Medicine, Nanjing, Jiangsu 210023 China; The Business Administration School, Chengdu University of TCM, Chengdu, Sichuan 610075 China; The Third Affiliated Hospital of Nanjing University of TCM, Nanjing, Jiangsu 210029 China

**Keywords:** Electro-acupuncture, Lifestyle modification, Simple obesity, Overweight, Comparative effectiveness research

## Abstract

**Background:**

Acupuncture is considered to be an effective and safe treatment for obese and overweight patients, although high-quality evidence regarding the effects of acupuncture on obesity are not conclusive. The aim of the current study is to investigate the effectiveness of electro-acupuncture plus lifestyle modification for treating obese and overweight patients, in comparison with lifestyle modification alone in China.

**Methods/Design:**

To compare the effectiveness of acupuncture plus lifestyle modification, a 2-armed, controlled trial with randomization using minimization will be conducted on 150 simple obesity and overweight patients, aged 18–50 years, for a 36-week study duration. All patients will be randomly assigned to one of two groups and will receive either acupuncture plus lifestyle modification or lifestyle modification alone. Outcomes will be evaluated at baseline and at 4 weeks, 8 weeks, and 12 weeks during treatment as well as at 6-week, 12-week, and 24-week follow-up. The primary endpoint is change of body mass index (BMI) during the 12th week. Secondary endpoints are body weight; waist-to-hip ratio; biochemical tests including serum cholesterol (TC), triglyceride (TG) and high-density lipoprotein (HDL) levels; and answers to the Short Form 36 (SF-36) and the Impact of Weight on Quality of Life Questionnaire-Lite Version (IWQOL-Lite). Statistical analyses will be based on the intention-to-treat (ITT) principle. The main endpoint will be analyzed by analysis of covariance (ANCOVA), and the objective outcome results will be analyzed by logistic regression analysis. To avoid potential confounding factors, additional sensitivity analyses will be conducted following these statistical analyses.

**Discussion:**

This trial is the first to compare the effectiveness of acupuncture plus lifestyle modification for treating obesity relative to lifestyle modification treatment alone by using a pragmatic study design. We hope that the results of this study will contribute to advancing the current methodology of acupuncture trials for obesity and will facilitate the application of useful acupuncture strategies in real-world clinical settings.

**Trial registration:**

ChiCTR-TRC-12002762. The date of registration is 31 October 2012.

## Background

Obesity, defined as a body mass index (BMI) greater than 28 kg/m^2^ in China, is a serious and prominent health issue worldwide. It contributes to multiple morbidities, including coronary heart disease, type 2 diabetes, polycystic ovary syndrome (PCOS), breast cancer, colon cancer, renal cancer, and many other serious pathologies [1–4]. Currently, obesity is estimated to affect over 396 million individuals and to cause 3.4 million deaths, 3.9 % of years of life lost, as well as 3.8 % of Disability-adjusted Life-Years (DALYs) across the globe [5, 6]. Moreover, the prevalence of obese and overweight adults in China has risen to 12.0 % and 30.6 %, respectively, during 2010 [7]. In response to the sharp increase of obese and overweight individuals, numerous obesity treatments are commonly used on these patients. However, pharmacological drugs for the management of obesity and being overweight, including orlistat, lorcaserin, and the combination of phentermine and topiramate, are constantly being debated due to their side effects, such as gastrointestinal and menstrual disorders, possible liver damage, and lack of long-term safety assurance [8–10].

Acupuncture, on the other hand, originating from China and established on the framework of traditional Chinese medicine (TCM), is one of the most important alternative and complementary therapies for treating numerous diseases. While acupuncture has been practiced worldwide, the lack of high-quality clinical evidence makes acupuncture a highly controversial therapy. Based on a series of clinical studies, which showed that acupuncture at acupoints was no more effective than acupuncture at non-acupoints, some researchers have wondered whether the effect of acupuncture is only due to the placebo effect or to bias. Recently, a meta-analysis including 18,000 randomized patients in high-quality randomized control trials has manifested novel evidence that acupuncture is superior to sham acupuncture and placebo for managing chronic pain [11]. Meanwhile, a meta-analysis containing 3013 individual patients has also indicated that acupuncture can significantly reduce average body weight (95 % confidence interval (CI) of 1.72 kg (0.50–2.93 kg)) and improve obesity rates (relative risk = 2.57; 95 % CI, 1.98–3.34) compared to lifestyle modification [12]. Furthermore, the latest systematic review has shown that acupuncture is more effective than placebo or lifestyle modification to reduce body weight and has similar effects as western anti-obesity drugs but with fewer adverse effects [13]. Thus, there is an increasing tendency to use acupuncture as an adjunctive therapy based on basic lifestyle modification treatment for obesity in both western and eastern countries.

Although acupuncture’s multiple benefits have been proved by many, convincing evidence regarding the use of acupuncture for treating obesity is still limited due to the low-quality methodologies and small sample sizes. Cho et al. [12] have summarized that two thirds of acupuncture trials on obesity, drawing from a total of 31 studies, are valued as having low Jadad scores. By performing a systematic review of acupuncture and Chinese medicine on obesity, Sui et al. [13] have concluded that studies conducted within China have a lower methodological quality than those outside of China. Sui et al. also have argued that the limitations in Chinese trials include a lack of information on blinding, methods of randomization, and reasons for discontinuation. Additionally, there was little confirmatory evidence regarding the types of acupuncture offered to obese patients. Various types of acupuncture, including manual acupuncture, electro-acupuncture, auricular acupuncture, auricular acupress, electro-acupuncture plus auricular acupress, and electro-acupuncture plus lifestyle modification, are applied for daily clinical obesity treatment. Although electro-acupuncture is more frequently performed in both clinical obesity treatment and obesity experiments [12–16], so far there is no convincing evidence supporting its effectiveness in treating obesity. Hence, two pivotal questions relating to electro-acupuncture for obesity treatment have been raised. First, does electro-acupuncture plus lifestyle modification show a better effect than conventional lifestyle treatment for treating obese or overweight patients in a high-quality randomized controlled trial in China? Second, is electro-acupuncture plus lifestyle modification treatment suitable for daily clinical obesity treatment?

To address these two questions, here we are proposing a pragmatic study design. Pragmatic trials, such as comparative effectiveness trials, have been widely employed to evaluate the effectiveness of an intervention applied for the usual clinical conditions [17–19]. The advantage of the pragmatic design is that it properly suits the essence of Chinese medicine and acupuncture for its complexity and pragmatism. With the aid of the comparative effectiveness trial design, acupuncturists can perform different treatments and determine personalized acupoints by themselves in acupuncture trials [20–22]; this offers flexibility for both acupuncturists and patients, with the benefit of the clinical effects of acupuncture according to TCM doctrine. Hugh et al. [23] have recently performed a comparative effectiveness trial to compare the effectiveness of acupuncture and counseling for treating depression. This research set a successful example for the use of pragmatic trials in clinical acupuncture studies.

Herein, we designed a comparative effectiveness trial to evaluate the effectiveness of electro-acupuncture plus lifestyle modification treatment for simple obesity and overweight patients.

## Aims

The main objective of this study is to investigate the effectiveness of electro-acupuncture plus lifestyle modification treatment for simple obesity and overweight patients (assessed by BMI and body weight) in comparison with lifestyle modification treatment alone.

## Methods

### Study design

A randomized, controlled, 2-armed trial using a parallel design with a 36-week study duration for each patient will be conducted to test 2 different hypotheses:

H_0_: acupuncture plus lifestyle modification = lifestyle modification (null hypothesis) versus H_1_: acupuncture plus lifestyle modification ≠ lifestyle modification (alternative hypothesis).

**Fig. 1:**
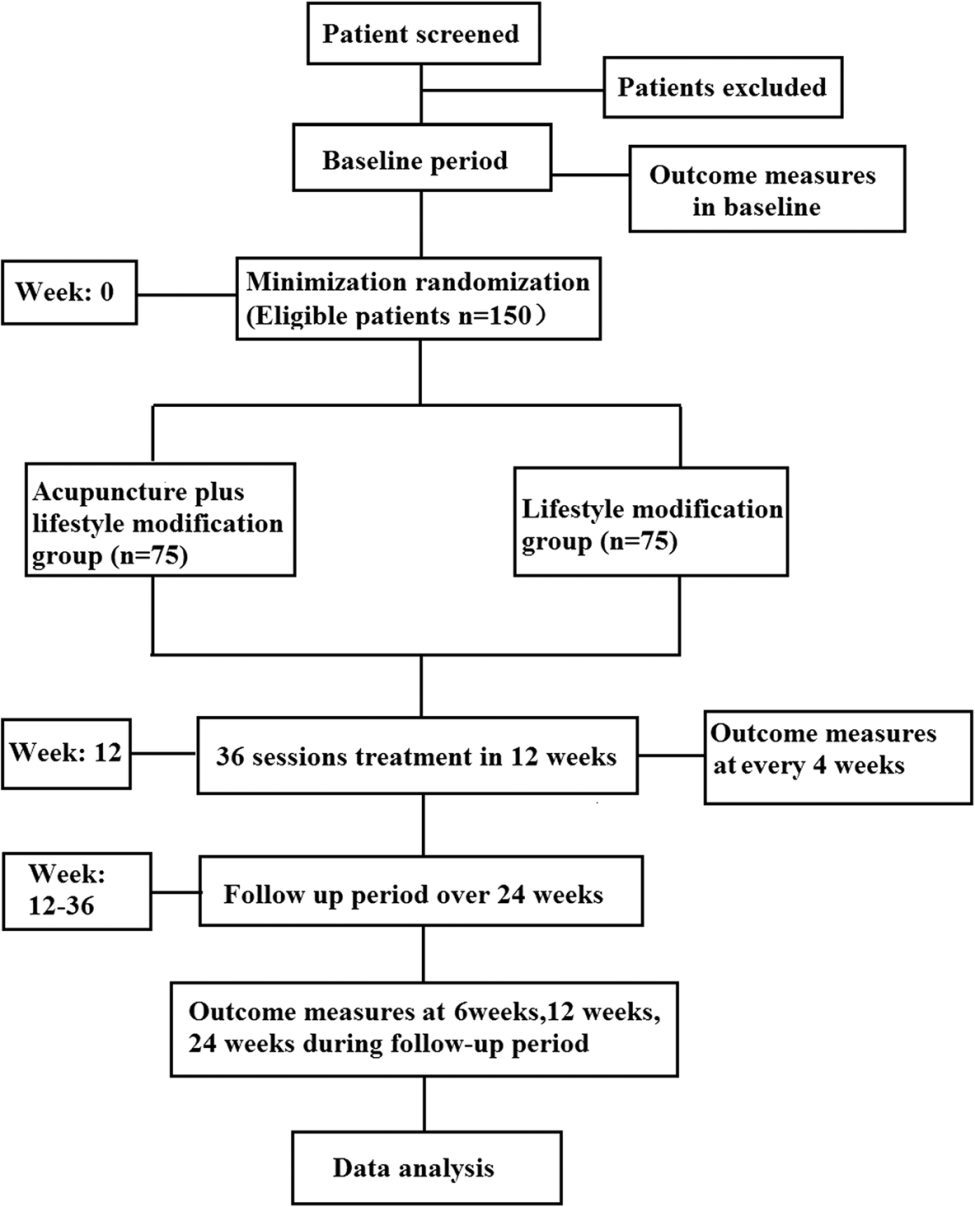
Flow chart of study

To test these hypotheses, patients will be randomized into one of two groups as shown in Fig. 1.

### Patients

To be included in this study, patients need to fulfill the following criteria: be male or female, aged 18–50 years old, and have a BMI ≥ 24 kg/m^2^, as diagnosed by their general physician in accordance with the guidelines of prevention and treatment for overweight and obesity in Chinese adults [24]. The patients should have failed previously to achieve their weight-loss goals through diet and exercise alone, and be willing to accept acupuncture and lifestyle modification after the explanation of risks and benefits regarding these two therapies. In addition, the ability to read and understand consent forms and questionnaires is required.

Patients will be excluded if they meet the following criteria: they have inherited obesity or secondary obesity; an endocrine disease such as a thyroid disorder, pituitary disorder, or sex gland disorder; heart disease such as arrhythmia, heart failure, myocardial infarction, or require a pacemaker; allergic or immune disease; kidney disease such as diabetic nephropathy, secondary focal segmental glomerulosclerosis, high amino transferases (alanine, aspartate > 50 U/L), or high serum creatinine (4.25 mg/dL); or are a pregnant or lactating woman. In addition, patients who have managed their weight for 1 month or have any other conditions unsuitable for this trial as evaluated by a general physician will be excluded.

All patients will be recruited in outpatient clinics of the Affiliated Hospital of Nanjing University of Chinese Medicine. Additional advertisements will be conducted in five universities in Nanjing, China. *The Handbook of Obesity* will also be printed and sent to all patients.

### Minimization randomization and blinding

Minimization randomization scheme will be performed at the Nanjing Good Clinical Practice (GCP) center through three different steps according to the extending Consolidated Standards of Reporting Trials (CONSORT) statement of Revised Standards for Reporting Interventions in Clinical Trials of Acupuncture (STRICTA) [25]: sequence generation, allocation concealment, and implementation.

#### Sequence generation

Patients will be randomly allocated to one of two groups in a 1:1 ratio using a minimization procedure controlled by the GCP center. Minimization is used to balance the severity of obesity, age, and gender between the two groups. The severity of obesity is divided into 4 levels, including overweight (24 ≤ BMI < 28), obesity I (28 ≤ BMI ≤ 32), obesity II (32 < BMI < 35), and obesity III (BMI ≥ 35) [26–29]. Once the severity of obesity, age, and gender of the patients are inputted in the online software, the minimization procedure will be implemented by a skilled statistician.

#### Allocation concealment

An Email or short message service message (SMS) detailing the random numbers and group assignment will be sent directly to the study assistant from the GCP center. Acupuncturists in this trial will not recruit patients, and the group assignment cannot be changed once it has been made.

#### Implementation

All patients who meet the inclusion criteria will be enrolled by one of two independent recruiters from the outpatient clinics of the study hospital and universities. After inclusion, a study assistant will send the patient’s information, including name, gender, numerical birthday, and BMI index, to the GCP center. A random allocation sequence will be generated by the GCP center computer when it receives the minimization information. Finally, the GCP center computer will send the random numbers and the group assignment back to the study assistant by Email or SMS.

#### Blinding

The acupuncturists and nutritionists will not be blinded due to the pragmatic trial design. The statistician will remain blinded from the identity of the two treatment groups until the end of the study.

## Intervention

### Electro-acupuncture plus lifestyle modification group

#### The acupuncture prescription

The acupuncture prescription was selected by three steps: first, literature articles and systematic reviews regarding ancient acupuncture treatment for obesity were collected [12, 13, 30–32]; the frequency of acupoints for obesity was analyzed. The stomach, spleen, *Ren* meridian, ST36, ST40, ST25, SP6, CV12, CV4, and CV6 are most frequently used in obesity treatment. Subsequently, the further acupuncture protocol was discussed and developed with experts from multiple disciplines, including professional acupuncture experts, acupuncturists, nutritionists, physicians, and statisticians. LI11, LI4, CV12, ST25, CV4, ST36, SP6, and ST44 were finally selected for use based on the literature, clinical experience, and TCM doctrine.

Specifically, semi-standard acupuncture treatment will be applied in this study. The acupuncturist who performs acupuncture on patients can choose additional acupoints beyond this main acupuncture prescription according to their TCM diagnoses and experiences. In addition, standard operating procedures (SOPs) of acupuncture will be constructed according to the Revised STRICTA guidelines [25].

#### Application of electro-acupuncture

Patients allocated to the acupuncture plus lifestyle group will receive electro-acupuncture treatment as follows: a total of 7 sterile disposable stainless steel needles (diameter: 0.25 mm; length: 40 mm, Hwatuo, Suzhou, China) will be punctured unilaterally at a depth of 25–35 mm in LI11, LI4, CV12, CV4, ST36, SP6, and ST44 on the patients, and performed alternately at the next treatment. Another 2 sterile disposable stainless steel needles will be punctured bilaterally at a depth of 25–35 mm in ST25 on the patients. Acupuncture manipulation will be used to achieve the *Deqi* sensation. Transcutaneous electro-acupuncture stimulation will be then conducted at acupoints for 30 minutes using a Han’s acupoint nerve stimulator (HANS-200, Nanjing, China). The stimulation frequency will be set at 2/15 Hz, and the intensity will vary from 0.1 mA to a maximum of 2.0 mA until the needle handle begins to tremble slightly. Acupuncture treatment will be performed 3 times per week according to the patient’s convenience, and a total of 36 acupuncture treatments will be performed on the patients in the acupuncture plus lifestyle modification group. All the acupuncturists in this trial are qualified TCM doctors. They have at least 8 years of acupuncture training experience and have a Master's degree in acupuncture.

In addition, patients allocate to the electro-acupuncture plus lifestyle modification group will also receive lifestyle modification treatment combined with electro-acupuncture treatment. The lifestyle modification treatment will be identical to that used for the lifestyle modification group below.

#### Lifestyle modification group

The lifestyle modification treatment was designed by experienced nutritionists, and the treatment protocol was established before the study began. Firstly, each patient’s lifestyle, including daily nutrient intake and exercise habit, will be collected using a dietary questionnaire [33, 34] and analyzed by a nutritionist at the beginning of the study. A face-to-face nutritional consultation will be subsequently arranged for each patient, in order to tailor an individualized lifestyle treatment. The possible goal of weight reduction for each patient will be set at this face-to-face nutritional consultation. Accordingly, a 24-hour food recall diary will be used to record mean nutrient intake during 24 hours for all the patients [35–37]. Three days of food recall diaries during each week will be collected from each patient within 3 months, for the purpose of estimating nutrition intake and self-monitoring dietary food intake. Individualized nutrition and activity advice will be given to each patient in terms of their weight-loss goals and food diaries during each week. In detail, according to Dietary Guidelines for Chinese Residents [38], it will be suggested that overweight and obese patients reduce at least 300–500 kcal of their daily nutrition intake, as well as increase by 8000–10,000 steps their daily activities during treatments. Finally, a total of 12 nutritional consultations will be given to all the patients enrolled into study within the 12-week treatment period. Specifically, social network software (QQ, WeChat) will be used to establish a social network among all the patients, promoting the persistence of treatment.

#### Outcomes

The primary outcome is the change of BMI during the 12th week after minimization. The formula for BMI is as follows:

BMI = mass (kg)/(height(m))^2^.

The BMI of all patients will be calculated at baseline; during treatment at 4, 8, and 12 weeks; and 6-week, 12-week, and 24-week follow-up [39]. The BMI reduction will be assessed by a blinded investigator from the Clinic of Nanjing University of Chinese Medicine.

The secondary outcomes are divided into objective outcomes and patient-centered outcomes. The body weight and the waist-to-hip ratio will be used as supplementary measures in combination with the BMI, and will be calculated at baseline; during treatment at 4, 8, and 12 weeks; and at 6-week, 12-week, and 24-week follow-up. In addition, TC, TG, and HDL levels also will be tested at baseline; during treatment at 4, 8, and 12 weeks to assess the metabolic changes [40]. For patient-centered outcomes, IWQOL-Lite and SF-36 are used to measure obesity-specific quality of life. The IWQOL-Lite questionnaire is a validated, obesity-specific measure, consisting of 31 items regarding the impact of obesity on physical function, self-esteem, sexual life, public distress, and work [41–43]. Patients will be asked to rank items related to obesity in 5 areas, ranging from 5 “always true” to 1 “never true”, by an independent assessor. The independent assessor will give a detailed explanation regarding every item of this questionnaire to patients before the treatment. All the patient-centered outcomes will be detected before treatment; during treatment at 4, 8, and 12 weeks; and at 6-week, 12-week, 24-week follow-up. Adverse events and serious adverse events will be recorded during the study to assess safety. By using a valid expectation questionnaire relating to acupuncture [44], participants’ expectations for acupuncture at baseline will also be assessed.

### Statistics

#### Sample size calculation

The sample size was determined prospectively as described in a previous study that used similar outcome measurements [45]. The following formula was used to estimate the sample size:

$$ n=\frac{{\left({u}_{\alpha }+{u}_{\boldsymbol{\beta}}\right)}^2\kern0.5em \left( 1+ 1/k\right){\sigma}^2}{\delta^2} $$

An independent statistician will monitor the study design from the beginning of the study and take responsibility for calculating the sample size and all data. Acupuncture treatment is reported to cause a 4.84 ± 7.38 (mean ± standard deviation) kg reduction of body weight compared to lifestyle modification treatment alone, according to a previously published systematic review [12]. This study was designed to have 80 % power and a significance level of 5 % for the purpose of discriminating differences between electro-acupuncture plus lifestyle modification and lifestyle modification alone. To achieve this, 64 patients will be included in each group and a total number of 150 patients will be enrolled, allowing for a 15 % dropout rate per group.

#### Analysis

The primary outcome is the change of BMI during the 12th week after minimization. To determine differences between the 2 groups after 12 weeks, analysis of covariance (ANCOVA) will be used to adjust for baseline values. Because there are two primary analyses (electro-acupuncture plus lifestyle modification versus lifestyle modification alone), Bonferroni correction will be used to address multiple testing, and a significance level of 0.05 will be used.

Statistical analyses of primary and secondary outcomes will be conducted as follows. First, all the analyses will be based on the intention-to-treat (ITT) principle. All patients, who will provide baseline data, will be included in the analyses, regardless of whether or not they complete the treatment or adhere to the protocol. Data distribution is expected to be normal, and skewed distribution data will be transformed prior to analysis. The statistical hypotheses are:

H_0_: acupuncture plus lifestyle modification = lifestyle modification (null hypothesis) versus H_1_: acupuncture plus lifestyle modification ≠ lifestyle modification (alternative hypothesis).

ANCOVA will be subsequently performed on BMI, body weight, and waist-to-hip ratio data as well as SF-36 and IWQOL-Lite. SF-36 and IWQOL-Lite will be transformed to continuous variables before analysis. Missing values will be addressed using the methods of last observation carried forward (LOCF). To address the results of objective outcomes, logistic regression analysis will be used to analyze dependent variables, including TC, HDL, and TG levels. These statistical analyses will be performed using SPSS 15.0 statistics software (SPSS Inc., Chicago, IL, USA) and SAS 9.0 (SAS Institute Inc., Cary, NC, USA).

To avoid potential confounding factors, additional sensitivity analyses will be conducted following these statistical analyses by using STATA (StataCorp, College Station, TX, USA) or R methods. Patient expectations also will be included as ordinal fixed factors in the sensitivity analyses. Full analysis set and the potential confounding factors relating to baseline differences will be used to calculate the results.

#### Safety monitoring

Routine tests, including blood, urine, and stool sample analyses, electrocardiogram, and abdominal B ultrasound, will be performed on all patients before minimization. These test results will be part of the inclusion and exclusion evidence screened by a general physician. Acupuncture adverse events are defined as bleeding, fainting, hematoma, serious pain, and local infection. All adverse events, as well as managing these events, will be carefully recorded during the treatment and in the follow-up phases.

#### Quality control

A diverse study group including the principal investigator, recruiters, acupuncturists, nutritionists, data manager, monitor, and patients will be established before the study. All of them contribute to the design of this study. Basic study training to understand the design, purpose, and basic information will be performed before the study. In addition, an independent data manager will be responsible for saving and managing the various data. A checklist including all the important aspects during the study will be made, and a trained monitor will check this checklist and report the quality assurance throughout the trial in accordance with the protocol. Acupuncturists and nutritionists will be free to conduct individualized treatment for each patient due to the pragmatic trial design. Therefore, a few procedures will be undertaken to inspect their treatment. Patient withdrawal and adverse events during the study will be recorded in detail. Regular team meetings will be held and fully documented.

#### Regulatory and ethics approval

This study was approved by the Chinese Ethics Committee of Registering Clinical Trials (ChiECRCT-2012012) and is in compliance with the common guidelines for clinical trials (Helsinki Declaration). The registration number of this trial is ChiCTR-TRC-12002762. All recruited patients will obtain and sign an informed consent form.

#### Resource

This study is supported by grants from the National Natural Science Foundation of China (number 81202741, number. 81273838) and the Research Fund for the Doctoral Program of Higher Education of China (number 20123237120009).

## Discussion

For the first time, this study will evaluate the effectiveness of a combined intervention including both acupuncture and lifestyle modification for treating obesity in comparison with lifestyle modification alone in a normal clinical setting. The aim of this study is to conduct a pragmatic trial which not only will improve the methodology of acupuncture trials on obesity in China but also develop beneficial acupuncture strategies for obesity treatment favoring real-life practical settings. This protocol is in accordance with the STRICTA [22], the Effectiveness Guidance Document (EGD) for acupuncture research [46] and the extension of the CONSORT statement for reporting pragmatic trials [18]. The checklist for Comparative Effectiveness Research (CER) relevant aspects for acupuncture clinical studies has been checked before the study begins.

The main challenge of this trial is to innovate a pragmatic study protocol uniting personalized acupuncture treatment with advanced research methodologies. To resolve this challenge, a dimensional study group, including a renowned acupuncture professor, skilled acupuncturists, nutritionists, clinical methodologists, physicians, and patients, was built before the study began. Each aspect of the study design was fully discussed and revised by this group. Since the populations of obese and overweight adults in China have climbed to 12.0 % and 30.6 %, respectively, both obese and overweight patients will be enrolled in this study, reflecting those who usually receive treatment for weight-loss in terms of a pragmatic design. Lifestyle modification is the cornerstone among all the obesity treatments currently available. We therefore employ acupuncture plus lifestyle modification as an intervention in this trial, facilitating the real-world clinical treatment of obesity. To evaluate patient-reported outcomes, the IWQOL-Lite questionnaire will measure the impact of obesity on dimensional aspects of life. This questionnaire has been made by researchers at Duke University and has been used successfully in many obesity trials [47–50]. To monitor the food intake of overweight and obese patients, a 24-hour food recall diary will be applied to record mean nutrient intake during 24 hours. This diary is a valid instrument for estimating nutrition intake and a beneficial tool for self-monitoring of dietary intake. It has been proved and performed in numerous nutrition researches [32–34]. Moreover, minimization will be conducted as a random procedure in this trial, avoiding possible confounding factors such as different obesity levels, gender, and age. Additionally, an Internet social network will be established and be used throughout the study, promoting the persistence of treatment for patients during treatment and follow-up phases.

As this trial refers to two clearly different treatments, blinding is hard to perform after minimization. Hence, a limitation of internal validity and multiple potential biases, including expectations of the patients and providers, may occur during this study. To minimize these potential biases, most of the outcomes in this trial are designed to be objective outcomes, such as BMI, body weight, waist-to-hip ratio, and biochemical tests.

In summary, this is a pragmatic study design with a focus on the effectiveness of acupuncture plus lifestyle modification for treating obesity. It is our hope that this study is a useful attempt to advance the methodology of acupuncture trials, and the results may disclosure novel evidence for the effectiveness of acupuncture on obesity.

## Trial status

This study is recruiting patients.

## Abbreviations

ANCOVA: analysis of covariance
BMI: body mass index
CER: Comparative Effectiveness Research
CI: confidence interval
CONSORT: Consolidated Standards of Reporting Trials
DALYs: Disability-adjusted Life-Years
EGD: Effectiveness Guidance Document
GCP: Good Clinical Practice
HDL: high-density lipoprotein
LOCF: last observation carried forward
SF-36: Short Form 36
ITT: intention-to-treat
IWQOL-Lite: Impact of Weight on Quality of Life Questionnaire
PCOS: polycystic ovary syndrome
SMS: short message service
SOPs: standard operating procedures
STRICTA: Standards for Reporting Interventions for Controlled Trials of Acupuncture
TC: serum cholesterol
TCM: traditional Chinese medicine
TG: triglyceride
